# Overexpression of zinc-α2-glycoprotein suppressed seizures and seizure-related neuroflammation in pentylenetetrazol-kindled rats

**DOI:** 10.1186/s12974-018-1132-6

**Published:** 2018-03-22

**Authors:** Ying Liu, Teng Wang, Xi Liu, Yuetao Wen, Tao Xu, Xinyuan Yu, Xin Wei, Xueying Ding, Lijuan Mo, Maojia Yin, Xinjie Tan, Lifen Chen

**Affiliations:** 1 Department of Neurology, The Second Affiliated Hospital of Chongqing Medical University, 74 Linjiang Road, Yuzhong District, Chonqing, 400010 China; 2 Department of Neurosurgery, The University-Town Hospital of Chongqing Medical University, Chongqing, 401331 China

**Keywords:** Zinc-α2-glycoprotein, Epilepsy, Neuroinflammation, TNFα, IL-6, TGFβ

## Abstract

**Background:**

Zinc-α2-glycoprotein (ZAG) is a 42-kDa protein reported as an anti-inflammatory adipocytokine. Evidences from clinical and experimental studies revealed that brain inflammation plays important roles in epileptogenesis and seizure. Interestingly, closely relationship between ZAG and many important inflammatory mediators has been proven. Our previous study identified ZAG in neurons and found that ZAG is decreased in epilepsy and interacts with TGFβ and ERK. This study aimed to investigate the role of ZAG in seizure and explore its effect on seizure-related neuroinflammation.

**Methods:**

We overexpressed *AZGP1* in the hippocampus of rats via adeno-associated virus vector injection and observed their seizure behavior and EEG after pentylenetetrazol (PTZ) kindling. The level of typical inflammation mediators including TNFα, IL-6, TGFβ, ERK, and ERK phosphorylation were determined.

**Results:**

The overexpression of *AZGP1* reduced the seizure severity, prolonged the latency of kindling, and alleviated epileptiform discharges in EEG changes induced by PTZ. Overexpression of *AZGP1* also suppressed the expression of TNFα, IL-6, TGFβ, and ERK phosphorylaton in PTZ-kindled rats.

**Conclusions:**

ZAG may inhibit TGFβ-mediated ERK phosphorylation and inhibit neuroinflammation mediated by TNFα and IL-6, suggesting ZAG may suppress seizure via inhibiting neuroinflammation. ZAG may be a potential and novel therapeutic target for epilepsy.

## Background

Zinc-α2-glycoprotein (ZAG) is a 42-kDa, soluble, secretory protein encoded by the *AZGP1* gene located on chromosome 7q22.1 [1, 2]. The structure and amino acid sequence of ZAG are highly homologous to proteins in the major histocompatibility complex class I (MHC-I) family, which has important function in immunity [3]. In our previous study, we found that both ZAG protein and *AZGP1* mRNA levels were significantly decreased in brain tissues of refractory TLE patients and pentylenetetrazol (PTZ)-kindled rats [4], but the role of ZAG in epilepsy and seizure is still unclear.

ZAG is known to be involved in many molecular pathways linked to epilepsy and seizure, and it can regulate many epilepsy- or siezure-related molecules, although its role has never been studied in epilepsy and/or seizure. *AZGP1* overexpression can inhibit the mammalian target of rapamycin (mTOR) pathway activity [5], and ZAG can inhibit transforming growth factor-β (TGFβ)-mediated vimentin expression and extracellular regulated protein kinase (ERK) phosphorylation [6]. Meanwhile, mTOR, TGFβ, ERK, and phosphorylated ERK (pERK) were known to play important roles in epilepsy and seizure. The mTOR pathway regulates various cellular processes involved in growth, metabolism, structure, and cell–cell interactions of neurons and glia [7]. Inhibiting mTOR by rapamycin can suppress seizure, delay seizure development, or prevent epileptogenesis [7]. Increase of TGFβ in neurons was proven to be involved in epileptogenesis via regulating dendrite growth and synaptogenesis [8, 9]. The level of pERK in refractory epilepsy patients is significantly higher than that in controls [10], indicating an increased ERK activity in epilepsy. Inhibiting sodium glucose co transporter 2 (SGLT2) has been proven to increase the level of ZAG via activating PPARγ [11], while activating PPARγ was confirmed to increase *AZGP1* mRNA [12]. Interestingly, both SGLT2 and PPARγ are known to participate in epileptogenesis, especially PPARγ was considered as a promising therapeutic target of epilepsy [13–15]. ZAG can also increase the level of mitochondrial uncoupling proteins (UCP) [16], and PPARγ-upregulated mitochondrial UCP2 expression can ameliorate neuronal death in the hippocampus following status epilepticus [17]. Interactions between ZAG and these epilepsy- or seizure-related molecules or pathways suggest a potential role of ZAG in epilepsy and/or seizure.

Evidences from clinical and experimental studies revealed that brain inflammation plays important roles in epileptogenesis [18]. ZAG was reported as one of the anti-inflammatory adipocytokines including adiponectin, omentin, SFRP5, vaspin, and interleukin-10 (IL-10) [19]. ZAG was reported to block transforming growth factor-β (TGFβ)-mediated extracellular regulated protein kinase (ERK) phosphorylation [7]. While TGFβ plays a significant role in inflammation, inhibition of TGFβ receptor 1 or TGFβ1 diminished TGFβ1-induced inflammation [20]. ERK pathway also plays an important role in the inflammatory response [21–24]. We have verified the interaction between ZAG and TGFβ or pERK previously [4]. Therefore, it is possible that ZAG may participate in the pathogenesis and pathophysiology of epilepsy via regulating TGFβ-mediated ERK phosphorylation. In addition, increase of various inflammation-related cytokines has been observed in epilepsy [25–27]. Pro-inflammatory cytokines, such as tumor-necrosis factor (TNFα) and interleukin-6 (IL-6), were increased in epilepsy patients and epilepsy models [28–32]. Interestingly, TNFα has been identified to reduce ZAG production [33], while the interaction between ZAG and IL-6 has not been found.

The role of ZAG in seizure and seizure-related inflammation has not yet been discussed. In this study, we injected adeno-associated virus (AAV) that overexpress *AZGP1* to the hippocampus of rats and investigated the effect of *AZGP*1 overexpression on PTZ kindling-induced seizures in rats by behavior tests and scalp electroencephalogram. To further explore the molecular mechanism by which ZAG affects seizure and seizure-related inflammation, the effect of *AZGP1* overexpression on pERK, total ERK (tERK), TGFβ, TNFα, and IL-6 were also measured in the hippocampus of PTZ-kindled rats and controls.

## Methods

### Experimental animals

Adult, male, 200–300 g, specific-pathogen-free Sprague–Dawley rats (Experimental Animal Center of Chongqing Medical University) were raised in a temperature and humidity-controlled room (temperature 27 °C, humidity 55–65%) with a 12-h light/12-h dark cycle (lights on 6:00 and off 18:00), and they were allowed free access to food and water. All rats were housed for 1 week before the experiment.

### Adeno-associated virus (AAV) vector construction and stereotaxic injections

A DNA sequence that amplifies *AZGP*1 expression was incorporated into the adeno-associated virus (AAV) vector (pHBAAV-CMV-ZsGreen) containing green fluorescent protein (GFP) sequence and was named AAV–*AZGP1*. The same AAV vector containing only GFP was used as a control and named as AAV–GFP. Both vectors were manufactured by Hanbio Biotechnology (Shanghai, China). The final titer was 1.35 × 10^12^ vector genomes/ml for AAV–*AZGP*1 and 1.5 × 10^12^ vector genomes/ml for AAV–GFP.

To verify the successful transfection of AAV, 60 rats were randomly divided into 4 groups and treated as following (*n* = 15 for each group): (1) controls: rats received stereotaxic injection of saline; (2) GFP group: rats received AAV–GFP injection; (3) *AZGP1*-3W group: rats received AAV–*AZGP1* injection and recovered for 3 weeks; (4) *AZGP1*-9W group: rats received AAV–*AZGP1* injection and recovered for 9 weeks. After injection, GFP fluorescence was observed in the hippocampus of rats from GFP group, *AZGP1*-3W group and *AZGP1*-9W group (*n* = 5 for each group) as described below. Quantitative real-time polymerase chain reaction (qrt-PCR) (*n* = 5 for each group) and western blot (*n* = 5 for each group) were also performed in controls, GFP group, *AZGP1*-3W group, and *AZGP1*-9W group as described below.

When performing stereotaxic injection, rats were anesthetized by intraperitoneal injections of pentobarbital (60 mg/kg) and then placed in a stereotaxia frame (RWD Life Science, Shenzhen, China). After disinfection, skin of the dorsal surface of rat skull was cut apart, and two parallel holes were created using dental drill in the skull. The stereotactic coordinates of bilateral hippocampus region were 3.0 mm posterior to bregma, 2.0 mm lateral to median line of the skull, and 2.8 mm deep beneath the skull. 4 μL AAV–*AZGP1* or AAV–GFP was injected into the hippocampus through a glass pipette at a speed of 0.2 μL/min. To prevent backflow of vectors, the pipette was kept in the hippocampus for an additional 5 min and then retracted steadily and slowly. For the controls, an equal volume of saline was injected in the same way. In most cases, the surgical procedure last about 45 min for each rat, and most rats recovered from anesthesia 1 h after surgery ending, subsequent injection of pentobarbital was not necessary in our experiment. Rarely, rats had respiratory failure after surgery was rescued using atropine (1 mg/kg, intraperitoneally).

### PTZ kindling

After the AAV transfection and *AZGP1* expression was verified successful, another 45 rats were randomly divided into three groups (*n* = 15 for each group): controls, *AZGP1* + PTZ group and GFP + PTZ group. Rats in *AZGP1* + PTZ group and GFP + PTZ group recovered for 3 weeks after AAV injection and then received intraperitoneal injection of PTZ (35 mg/kg, Sigma-Aldrich, St. Louis, USA) for 28 days daily. After each injection of PTZ, all rats were observed for 30 min in plastic cages to assess and record the seizure severity according to a modified Racine scale as follows [34]: grade 0, no response; grade 1, facial myoclonus; grade 2, head nodding; grade 3, forelimb clonus; grade 4, rearing and severe forelimb clonus; grade 5, rearing, falling, and severe forelimb clonus. Controls (*n* = 15) received equal amount of saline instead of AAV and PTZ. Rats that exhibited stage 4 or 5 seizures on 3 consecutive days were considered to be fully kindled. Latency was defined as the days between the first PTZ injection and fully kindling. PTZ injection was conducted between 13:00 and 16:00 in order to minimize possible complicating effects on the behavior of the animals’ circadian rhythms.

### Scalp EEG recording

Scalp EEG was performed at the last time of PTZ injection. Two unipole scalp electrodes were placed on bilateral temporal skin of rats. After the place of electrodes, rats were allowed to move freely in plastic cages. EEG baseline was recorded for approximately 5 min before the injection of PTZ or saline, then EEG was recorded for at least 30 min using the nicolet vEGG system (natus, USA) and analyzed using nicoleton bms 5000 (natus, USA). The parameters of EEG are set as follows: filtering 30 Hz, paper speed 30 mm/s, and sensitivity 70 μV/mm. All remaining rats that received PTZ injection for 28 days were sacrificed for further research.

### Tissue processing

Rats were deeply anesthetized by intraperitoneal injection of pentobarbital (60 mg/kg). For western blot and quantitative real-time polymerase chain reaction (qrt-PCR), rat brains were removed and stored at − 80 °C. For GFP fluorescence observation, the rat brains were removed after perfusion with saline and 4% paraformaldehyde in phosphate-buffered saline (PBS) by cardiac puncture via the left ventricle. For comparing level of ZAG protein and *AZGP1* mRNA 3 and 9 weeks after AAV injection, some rat brain tissues were stored for 6 weeks at − 80 °C before western blot and qrt-PCR examination. The rest rat brain tissues were examined in 2 weeks after removal.

### qrt-PCR

Total RNA was extracted from the brain tissue using RNAiso plus (Takara, Dalian, China) and was reverse transcribed into complementary deoxyribonucleic acid (cDNA) with the Applied Biosystems Veriti-Well Thermal Cycler (Thermo, Wilmington, USA) using the PrimeScript RT reagent Kit with genome DNA Eraser (Takara, Dalian, China) following the manufacturer’s instructions. Briefly, 2 μg total RNA was mixed with 4 μl Reverse Transcriptase and 0.5 μg oligo (dT) primer and incubated at 37 °C for 15 min, and the reaction was then terminated at 85 °C for 5 s. Each qrt-PCR reaction contained 2 μl cDNA, 0.8 μl forward primer, 0.8 μl reverse primer, 6.4 μl DEPC water, and 10 μl SYBR Premix ExTaq II (Takara, Dalian, China). The PCR protocol consisted of an initial denaturation step at 95 °C for 30 s, followed by 40 cycles of amplification at 95 °C for 5 s and at 60 °C for 34 s, and then terminated at 95 °C for 15 s. Melting curve analyses were also performed (65.0 to 95.0 °C, 0.5 °C increments for 5 s). The relative gene expression levels in the hippocampus of PTZ-kindled rats were calculated using the 2^−ΔΔCt^ method [35]. The primer sequences for rat were as follows: *AZGP1*: forward 5′-TTCAAGCCACCGCATTTCTC-3′, reverse 5′-TCCTTCTCCCAGTCCTCCATTC-3′. GAPDH: forward 5′-ACGGTCAGGTCATCACTATCG-3′, reverse 5′-GGCATAGAGGTCTTTACGGATG-3′.

### Observation of fluorescence of GFP

The fixed brain tissues were successively immersed in 20 and 30% sucrose solution for 48 h (24 h per solution) and sliced into 10-μm-thick frozen sections. Finally, the sections were mounted using 80% glycerol. Images were collected using laser scanning confocal microscopy (Nikon 1R, Japan).

### Western blot

Brain tissue was homogenized in RIPA lysis buffer (Beyotime, Haimen, China) containing proteinase inhibitor mixture and phosphorylase inhibitor mixture and centrifuged at 12000 rpm, 4 °C for 25 min. The protein concentrations in the supernatant were determined using a BCA Protein Assay Kit (Beyotime, Haimen, China). The extracted total proteins were mixed with 5× sodium dodecylsulfate (SDS) loading buffer and boiled for 5 min. Equal amounts of total protein (80 μg/lane) were separated by SDS-polyacrylamide gel electrophoresis (PAGE) and then transferred onto polyvinylidene fluoride (PVDF) membranes (Immobilon, Merck Millipore, Darmstadt, Germany). The membranes were blocked with 5% BSA at room temperature for 2 h and then incubated with the anti-ZAG antibody (1:400, Santa Cruz, USA), anti-TNFα antibody (1:500, Bosterbio, USA), anti-IL6 antibody (1:500, Bosterbio, USA), anti-pERK antibody (1:2000, Cell Signaling Technology, Danvers, USA), anti-tERK antibody (1:2000, Proteintech, Wuhan, China), anti-TGFβ antibody (1:1000, Proteintech, Wuhan, China), or anti-glyceraldehyde 3-phosphate dehydrogenase (GAPDH) antibody (1:3000, Proteintech, Wuhan, China) at 4 °C overnight. After washed with tris-buffered saline with Tween-20 (TBST), the membranes were incubated with the horseradish peroxidase-conjugated rabbit anti-mouse antibody (1:3000, Abcam, Cambridge, UK) or mouse anti-rabbit antibody (1:3000, Abcam, Cambridge, UK) at room temperature for 1 h and washed again. Immunoreactivity was visualized using chemiluminescence substrate kit (Beyotime, Haimen, China) and quantified by densitometric scanning with the Fusion-FX7 system (Vilber Lourmat, Collégien, France). The mean optic density (OD) was normalized by GAPDH.

### Statistical analysis

The results were expressed as mean ± standard deviation (SD). SPSS 20.0 (IBM, Armonk, USA) and GraphPad prism 6.01 (GraphPad software, La Jolla, USA) were used for data analysis and graph drawing. Mann–Whitney *U* test was used to compare the differences of Racine’s scores between AAV–*AZGP1* group and AAV–GFP group. Student’s *t* test was used to compare the differences of latency between AAV–*AZGP1* group and AAV–GFP group. One-way ANOVA with Bonferroni or Dunnett’s T3 post hoc analysis was used to compare the level of proteins between the three groups. *p* < 0.05 (two tailed) was regarded statistically significant.

## Results

### The transfection of AAV and the overexpression of *AZGP1*

**Fig. 1:**
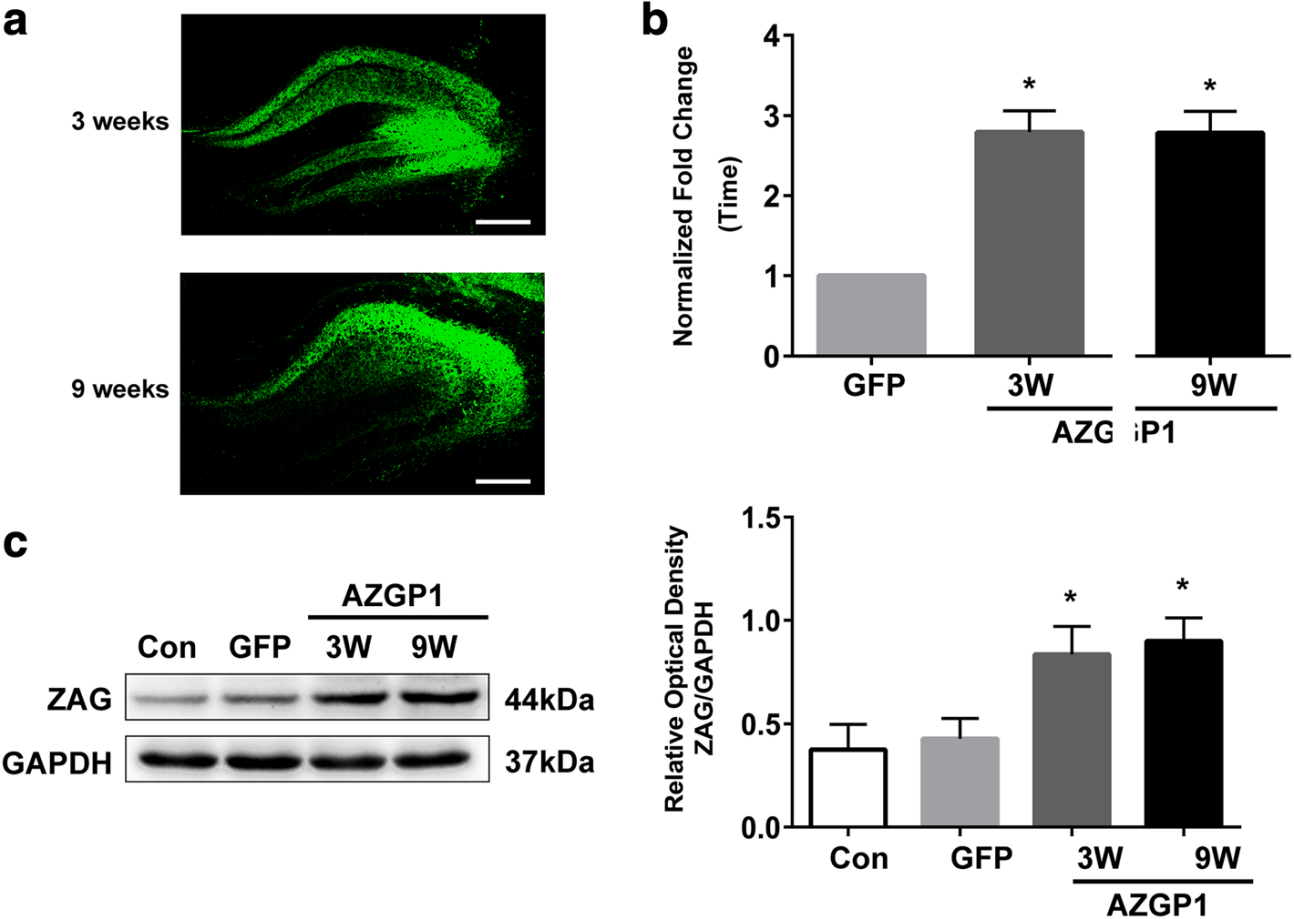
Expression of GFP and *AZGP*1 after injection of AAV vectors. **a** Fluorescent images showing GFP expression in the hippocampus of rats 3 and 9 weeks after AAV injection. The scale bar = 500 μm. **b** qrt-PCR showed increased *AZGP1* mRNA level in the hippocampus of rats in AAV–*AZGP1* group compared to AAV–GFP group 3 and 9 weeks after AAV injection. **c** Western blots showed increased ZAG protein level in the hippocampus of rats in AAV–*AZGP1* group compared to AAV–GFP group 3 and 9 weeks after AAV injection. Optic density was normalized by GAPDH. *n* = 5 for each group, *p < 0.05

To confirm the efficiency and stability of AAV-induced *AZGP1* expression, we detected GFP distribution and measured the level of *AZGP1* mRNA and ZAG protein in rat hippocampus 3 weeks and 9 weeks after AAV injection. The GFP-positive cells in the CA3 region of hippocampus were visualized (Fig. 1a). The level of *AZGP1* mRNA (Fig. 1b) and ZAG protein (Fig. 1c, d) was significantly increased on 3 and 9 weeks in *AZGP*1 group compared to GFP group. There was no difference of *AZGP1* mRNA (Fig. 1b) and ZAG protein (Fig. 1c, d) levels between 3 and 9 weeks in *AZGP1* group, suggesting a steady expression of *AZGP1*. In addition, no significant difference of *AZGP1* mRNA (Fig. 1b) and ZAG protein (Fig. 1c, d) level was found between GFP group and controls, suggesting no effect of AAV vectors on *AZGP1* mRNA and ZAG protein expression.

### Overexpression of *AZGP1* suppresses seizures in PTZ-treated rats

**Fig. 2:**
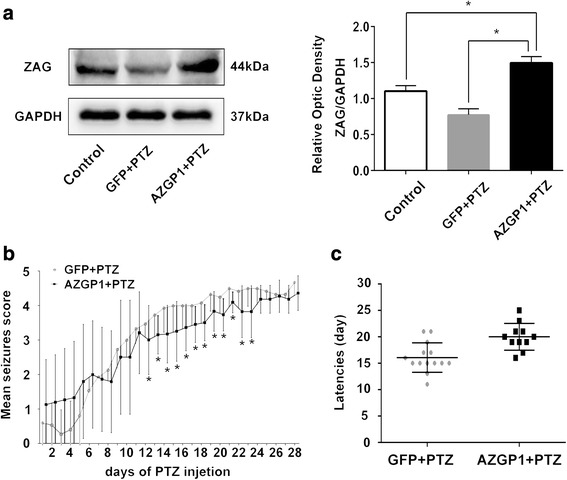
Overexpression of *AZGP1* suppressed seizure in PTZ-treated rats. **a** ZAG protein level in rats of *AZGP1* + PTZ group was significantly increased compared to GFP + PTZ group and controls. **b** Rats in *AZGP1* + PTZ group had significantly alleviated seizure severity during 12th to 23rd day of PTZ injection compared to GFP + PTZ group, while after 24th day of PTZ injection, there is no difference in seizure severity. **c** Overexpression of *AZGP*1 significantly prolonged the latency of PTZ kindling. *n* = 15 for each group, *p < 0.05

**Fig. 3:**
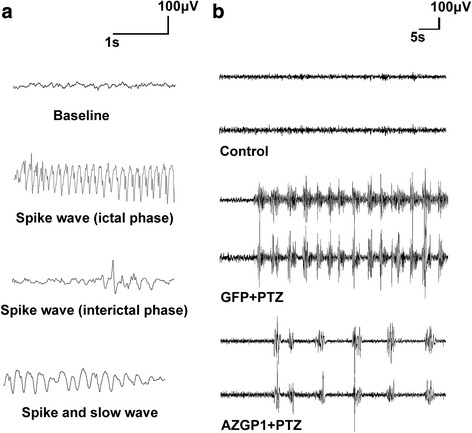
Scalp electroencephalogram (EEG) changes in *AZGP1* + PTZ group and GFP + PTZ group after PTZ kindling. **a** Typical EEG record of seizure. **b** Representative EEG record among the three groups of rats. Rats in *AZGP1* + PTZ group had significantly decreased frequency and amplitude of epileptiform spike waves in EEG compared to GFP + PTZ group

ZAG protein level in rats of *AZGP1* + PTZ group (OD 1.496 ± 0.086) was significantly higher than in the GFP + PTZ group (OD 0.778 ± 0.080) and controls (OD 1.104 ± 0.074) (*n* = 5, *p* = 0.0001, degrees of freedom (df) = 12, one-way ANOVA) (Fig. 2a). Rats in *AZGP1* + PTZ group had significantly milder seizure severity (Fig. 2b) before being fully kindled and longer latency (Fig. 2c) compared to rats in GFP + PTZ group (latency: *AZGP1* + PTZ 16.07 ± 2.786, *n* = 14 vs. GFP + PTZ 20.00 ± 2.530, *n* = 11, *p* = 0.0014, df = 23, Student’s *t* test). In addition, scalp EEG results showed significant decrease in the frequency and amplitude of seizure spike wave in *AZGP1* + PTZ groups compared to GFP + PTZ group (Fig. 3).

### Overexpression of *AZGP1* decreased the level of TGFβ and pERK in PTZ-kindled rats without affecting total ERK level

**Fig. 4:**
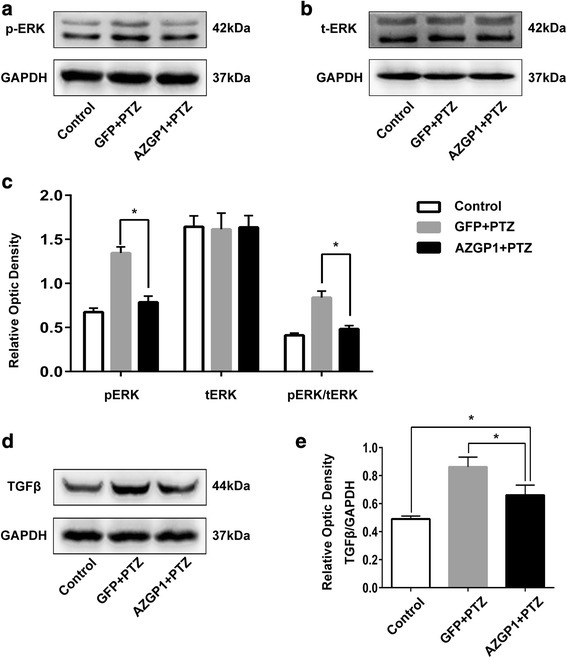
Overexpression of *AZGP1* decreased the level of TGFβ and pERK in PTZ-kindled rats without affecting total ERK level. **a**, **b** Representative western blot bands of p-ERK and t-ERK in hippocampal tissues of rats. **c** Western blot showed GFP + PTZ group had significantly increased level of p-ERK and p-ERK/t-ERK ratio in hippocampal tissues compared to controls, and rats in *AZGP1* + PTZ group had significantly decreased the level of pERK and pERK/tERK ratio compared to GFP + PTZ group. The level of tERK in hippocampal tissues is of no significant difference among the three groups. **d** Representative western blot bands of TGFβ in hippocampal tissues of rats. **e** Western blot showed increased level of TGFβ in the hippocampal tissues of rats in GFP + PTZ group compared to controls, and rats in *AZGP1* + PTZ group had significantly decreased the level of TGFβ compared to GFP + PTZ group. Optic density was normalized by GAPDH, *n* = 5 for each group, *p < 0.05

To explore the possibility that ZAG affects seizure via TGFβ-mediated ERK signaling pathway, we measured the level of pERK, total ERK, and TGFβ in the hippocampus of rats in the three groups using western blot. The level of pERK (Fig. 4a, c), pERK/total ERK ratio (Fig. 4c), and TGFβ (Fig. 4d, e) was significantly increased in both *AZGP1* + PTZ and GFP + PTZ groups compared to controls, while rats in *AZGP1* + PTZ group had decreased pERK (Fig. 4a, c), pERK/total ERK ratio (Fig. 4c), and TGFβ (Fig. 4d, e) compared to rats in GFP + PTZ group (pERK: control 0.674 ± 0.045, GFP + PTZ 1.344 ± 0.071, *AZGP1* + PTZ 0.787 ± 0.070, *p* = 0.0001, df = 12, one-way ANOVA); (pERK/total ERK ratio: control 0.410 ± 0.026, GFP + PTZ 0.840 ± 0.073, *AZGP1* + PTZ 0.482 ± 0.040, *p* = 0.0001, df = 12, one-way ANOVA); (TGFβ: control 0.490 ± 0.021, GFP + PTZ 0.862 ± 0.070, *AZGP1* + PTZ 0.660 ± 0.072, *p* = 0.001, df = 12, one-way ANOVA), (*n* = 5 for each group). In addition, there was no significant difference in total ERK level between the three groups (Fig. 4b) (control 1.642 ± 0.123, GFP + PTZ 1.614 ± 0.183, *AZGP1* + PTZ 1.636 ± 0.133, *p* = 1.000, df = 12, one-way ANOVA).

### Overexpression of *AZGP1* suppressed the increase of TNFα and IL-6 in PTZ-kindled rats

**Fig. 5:**
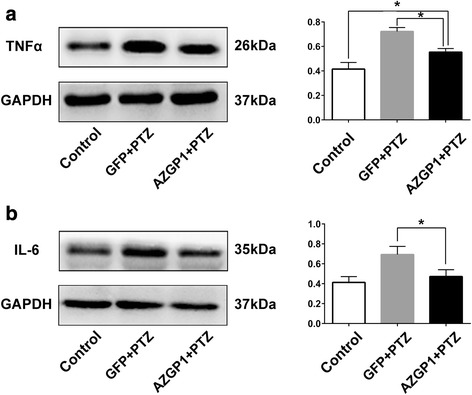
Overexpression of *AZGP1* suppressed the increase of TNFα and IL-6 in PTZ-kindled rats. **a** Representative western blot bands and expression level of TNFα in the hippocampal tissues of rats. Western blot showed increased level of TNFα in the hippocampal tissues of rats in GFP + PTZ group compared to controls, and rats in *AZGP1* + PTZ group had significantly decreased the level of TNFα compared to GFP + PTZ group. **b** Representative western blot bands and expression level of IL-6 in the hippocampus of rats. Western blot showed increased level of IL-6 in the hippocampal tissues of rats in GFP + PTZ group compared to controls, and rats in *AZGP1* + PTZ group had significantly decreased the level of IL-6 compared to GFP + PTZ group. Optic density normalized by GAPDH, *n* = 5 for each group, *p < 0.05

To assess the inflammatory state of the hippocampus, two typical pro-inflammatory cytokines, TNFα and IL-6, were measured using western blot in the hippocampus of *AZGP*1 + PTZ and GFP + PTZ group rats and controls. Hippocampal TNFα (Fig. 5a) level was increased in *AZGP*1 + PTZ and GFP + PTZ groups compared to controls, while in *AZGP*1 + PTZ group, TNFα (Fig. 5a) was significantly decreased compared to GFP + PTZ group (TNFα: control 0.415 ± 0.055, GFP + PTZ 0.722 ± 0.032, *AZGP*1 + PTZ 0.554 ± 0.029, *p* = 0.001, df = 12, one-way ANOVA). Hippocampal IL6 level was significantly decreased in *AZGP*1 + PTZ group compared to GFP + PTZ group (IL6: *AZGP1* + PTZ 0.472 ± 0.068, GFP + PTZ 0.692 ± 0.081, *p* = 0.001, df = 12, one-way ANOVA) while there is no statistical significance between *AZGP*1 + PTZ group and control (IL6: *AZGP1* + PTZ 0.472 ± 0.068, control 0.413 ± 0.058, *p* = 0.609, df = 12, one-way ANOVA) (Fig. 5b).

## Discussion

In our previous study, we had found that the level of *AZGP*1 mRNA and ZAG protein was significantly decreased in the neocortex of refractory epilepsy patients as well as in the hippocampus and neocortex of PTZ-kindled rats compared to controls [4]. However, the specific role of ZAG in epilepsy and/or seizure remains unclear.

In this study, behavior tests showed that overexpression of *AZGP1* in the hippocampus prolonged the latency of PTZ kindling and alleviated the seizure severity in PTZ-treated rats. Similarly, in scalp EEG recording, decreased frequency and amplitude of spike wave were also identified in AAV–*AZGP1* group. These results indicate a protective role of ZAG against seizure. Interestingly, we found that the seizure severity score of AAV–*AZGP1* group was significantly lower than AAV–GFP group during 12–23 days of PTZ treatment, while after the 24th day of PTZ treatment, the seizure severity was of no difference between the two groups, indicating that overexpression of ZAG can delay but not fully prevent the kindling. Moreover, on the 28th day of PTZ treatment, although the seizure severity of rats in both AAV–*AZGP1* group and AAV–GFP group were similar, the scalp EEG recording demonstrated decreased frequency and amplitude of spike waves in AAV–*AZGP1* group compared to AAV–GFP group. This result indicates that although rats in the two groups had similar seizure severity in behavior, their epileptiform discharge in brain is different. It is possible that overexpression of ZAG may have protective effect on seizure even if the rats are fully kindled. Although ZAG only delayed PTZ kindling in this study, it also alleviated the epileptiform discharges after the rats were fully kindled. It is possible that ZAG may prevent seizure in the early stage of kindling and has protective effect in the late stage of kindling, indicating a possible role of ZAG in different stage of epileptogenesis or seizure. This is the first study on the role of ZAG in seizure; further study is needed to explore its effect on seizure and mechanism.

ZAG was reported as an anti-inflammatory adipocytokine [24]. Clinical and experimental evidences have revealed that brain inflammation plays an important role in epileptogenesis [23]. The most extensively studied prototypical inflammatory cytokines in the central nervous system are TNFα and IL-6 [36–38]. The enhanced production of pro-inflammatory cytokines, including TNFα and IL-6, were known to play pro-epileptic roles in the brain [39]. The dynamic modulation of inflammatory processes has potential to be a novel therapeutic strategy for pharmacologic treatment to control seizures, delay disease progression, or retard epileptogenesis [40]. Moreover, overexpression of cytokines such as TNFα or IL-6 results in age-dependent increase of seizure susceptibility and spontaneous seizures [41, 42]. In our study, overexpression of *AZGP*1 attenuated the increase of TNFα and IL6 induced by PTZ kindling. Interestingly, TNFα has been identified to reduce ZAG production [33]. This is suggesting that there may be a circuit or feedback regulation mechanism between ZAG and TNFα. Therefore, ZAG may prevent seizure and protect the brain via alleviating neuroinflammation. This is the first time that the relationship between ZAG and IL-6 was identified.

We had previously verified the interaction between ZAG and TGFβ or p-ERK in the hippocampus of rats [4]. In this study, we found that overexpression of ZAG could decrease the level of TGFβ and ERK phosphorylation. Human recombinant ZAG was found to specifically block TGFβ-mediated ERK phosphorylation [6]. And TGFβ and p-ERK/ERK ratio were upregulated in patients with refractory epilepsy [8, 10]. TGFβ can promote epileptogenesis via upregulating IL-6 [43] and inhibiting TGFβ by losartan can suppress epileptogenesis [44]. ERK activation induced by its phosphorylation is known to cause seizure by activating *N*-methy-d-aspartate (NMDA) receptors [45, 46]. In addition, TGFβ and ERK are known as inflammatory mediators [20–24]. Thus, it is possible that ZAG may prevent seizure via inhibiting TGFβ-mediated ERK signaling pathway and alleviate inflammation induced by seizure. As the effect of ZAG on TGFβ and ERK has not been investigated in epilepsy and/or seizure before, further study is needed to clarify the existence of ZAG–TGFβ–ERK pathway and its specific role in seizure and epilepsy.

Many studies showed that various inflammatory cytokines are associated with seizure susceptibility [47–50]. Overexpressing IL-6 or TNFα can decrease seizure threshold and exacerbate seizure-induced neuronal loss [51, 52]. Inflammatory signaling is also known to worsen the loss of GABAergic neurons in the hippocampus and thus resulted in an increased susceptibility for seizure [53]. TNFα have also been associated with the regulation of seizure duration in amygdala-kindled rats [54]. In our study, overexpression of *AZGP1* in the hippocampus prolonged the latency of PTZ kindling and attenuated PTZ kindling-induced increase of TNFα and IL-6. This result suggests that overexpression of *AZGP1* may decrease seizure susceptibility in PTZ-treated rats, and this effect of overexpression of *AZGP1* may possibly be attributed to its inhibition of neuroinflammation. This is the first study relating ZAG to neuroinflammation in seizure; further study is needed to explore the relationship between ZAG and neuroinflammation in seizure. This study is a preliminary discuss on the role of ZAG in seizure, but more specific mechanism, such as ZAG–TGFβ–ERK pathway and the effect of ZAG on NMDA/AMPA/GABA receptors, still needs to be further researched. Furthermore, researches on the role of ZAG deficiency in epilepsy and/or seizure are also needed.

## Conclusion

Our study found that overexpression of *AZGP1* delayed PTZ kindling, alleviated seizure and epileptiform discharges, inhibited TGFβ-mediated ERK phosphorylation, and decreased TNFα and IL-6 in PTZ-treated rats. Our study indicated that ZAG may suppress seizure via inhibiting neuroinflammation. As an anti-inflammatory cytokine, ZAG may be a novel target for research and clinical treatment of seizure and possibly for epilepsy.

## Abbreviations

AAV: Adeno-associated virus
cDNA: Complementary deoxyribonucleic acid
EEG: Electroencephalography
ERK: Extracellular regulated protein kinase
GAPDH: Glyceraldehyde 3-phosphate dehydrogenase
GFP: Green fluorescent protein
IL-10: Interleukin-10
IL-6: Interleukin-6
MHC-I: Major histocompatibility complex class I
mTOR: Mammalian target of rapamycin
NMDA: *N*-methy-d-aspartate
OD: Optic density
PAGE: Polyacrylamide gel electrophoresis
PBS: Paraformaldehyde in phosphate-buffered saline
pERK: Phosphorylated extracellular regulated protein kinase
PPARγ: Peroxisome proliferator-activated receptor gamma
PTZ: Pentylenetetrazol
PVDF: Polyvinylidene fluoride
qrt-PCR: Quantitative real-time polymerase chain reaction
SD: Standard deviation
SDS: Sodium dodecylsulfate
SGLT2: Sodium–glucose cotransporter 2
TBST: Tris-buffered saline with Tween-20
TGFβ: Transforming growth factor-β
TNFα: Tumor-necrosis factor
UCP: Uncoupling proteins
ZAG: Zinc-α2-glycoprotein
